# Molecular simulation of water vapor outgassing from silica nanopores

**DOI:** 10.1007/s10404-015-1583-3

**Published:** 2015-05-05

**Authors:** Junghan Kim, Arjan J. H. Frijns, Silvia V. Nedea, Anton A. van Steenhoven

**Affiliations:** Department of Mechanical Engineering, Eindhoven University of Technology, PO Box 513, 5600 MB Eindhoven, The Netherlands

**Keywords:** Outgassing, Silica nanopore, Water, Molecular dynamics

## Abstract

The outgassing problem is solved numerically by molecular dynamics. A slit-shaped nanopore consisting of cavity and channel is built with an implicit tabulated wall potential that describes the water–silicon/silica interaction. A flexible three-point water model is used for the simulation. The effects of varying the system temperature, outlet pressure, geometry, and materials of the nanopore on the outgassing rate are investigated. The results show that the temperature plays an important role in the outgassing rate, while the effect of the outlet pressure is negligible as long as it is in the high to medium vacuum range. The geometry of the channel also has an influence on the outgassing rate, but not as much as the surface material. Three different types of silica materials are tested: silicon, silica-cristobalite (hydrophilic material), and silica-quartz (super hydrophilic material). The fastest outgassing rate is found for a silicon nanopore. It is also found that a thin water film is formed on the surface of the silica-quartz nanopore. This material shows hardly any outgassing of water.

## Introduction

Outgassing refers to gaseous emissions from solids; these gases have been reported to be adsorbed previously (Jousten 1999). It is usually enhanced by exposure to high operating temperatures and/or low external pressures. Outgassing effects can be often found in cracks of solid surfaces or in small cavities of MEMS/NEMS structures.

Outgassing can create a problem for the wafer-level packaging. Wafer-level packaging is essential for miniaturization and high-level integration in the semiconductor industry. It allows for the encapsulation of MEMS or integrated circuits (IC), before the standard IC packaging process takes place. Wafer bonding is a key technology for wafer-level packaging. By bonding a cap wafer to a wafer with MEMS, the mechanical structures can be sealed in a package and protected against the outside environment. However, unwanted gasses are sometimes created within the sealed microstructures during or after wafer bonding (Gao et al. 2012). Outgassing forms pressure, which may destroy the vacuum in the sealed microcavity. This can degrade the reliability of a MEMS/NEMS device, and it can lead to a failure of the device (Charvet et al. 2013). Outgassing sometimes creates a problem during the wafer bonding process. Fusion bonding of silicon is a very commonly used wafer bonding technology due to its simplicity and high bond strength (Lindroos et al. 2009). During this bonding process, wafer surfaces are brought into contact (pre-bonded) in vacuum condition followed by high temperature annealing to strengthen the bond. Outgassing of water molecules, remaining in the wafer before the pre-bonding stage, is found to be the main reason for bonding failures since it can lead to bubbles on the wafer surfaces (Gao et al. 2012).

Another problem of outgassing is that it can be a source of contamination to the vacuum chamber when ultrahigh vacuum (UHV) is necessary. UHV may be roughly defined as vacuum pressures below $10^{-8}$ mbar, and it is becoming more important since it is highly required for extreme ultraviolet (EUV) lithography. EUV lithography is one of the most promising methods for next-generation device manufacturing for semiconductor industry (Madey et al. 2006). A study of water outgassing is important since water is a dominant background gas in vacuum processing chambers (Madey et al. 2006). It can cause contamination and oxidation on multilayer reflecting optics surfaces. It will not only reduce system throughput because of the associated reduction in EUV reflectivity, but also introduce wavefront aberrations that compromise the ability to print uniform features (Madey et al. 2006).

Since outgassing molecules are a source of impurities in the semiconductor industry and cause MEMS/NEMS device failures, it is necessary to understand the nature of outgassing happening in micro- and nanosystems in order to control it.

Through many outgassing studies (Ishimaru et al. 1992; Ishikawa and Odaka 1990; Ishikawa et al. 1991; Ishikawa and Yoshimura 1995), it is well known that temperature and pressure are dominant factors for the outgassing process (Jousten 1999). These phenomena are well described by continuum flow models. However, vacuum industries are interested whether other factors play important role in the last stage of outgassing process when continuum models break down. Considering the great complexity of microfabrication processes, the understanding of the last stage of outgassing behavior also becomes a very complex task; it is dependent on temperature, outlet pressure, geometry, surface materials, etc. The influence of all these parameters has to be understood and controlled.

In order to understand the outgassing of water molecules in cracks of the surfaces or in MEMS or NEMS, we simulate the last stage of outgassing of water molecules from silicon/silica nanopores by using a molecular dynamics method.

## Method

In nanosystems, the Navier–Stokes equation breaks down and different particle-based models, including kinetic model equations, lattice Boltzmann method, Direct Simulation Monte Carlo (DSMC), and molecular dynamics (MD) have to be used.

DSMC is often used for micro- and nanochannel flow simulations because of its accurate simulation results for high Knudsen regimes and relatively fast computation speed compared to MD. However, DSMC also has some limitations. The diffusion coefficient is one of the main parameters determining the outgassing rates. But in most DSMC models other than extended models such as the GSS model (Fan 2002), the diffusion coefficients are less accurately computed for polar molecules such as ammonia and water vapor. It is also difficult to model attractive gas–surface interactions. Since sorption phenomena are another main factor determining the outgassing rate, it is necessary to have reliable gas–surface interaction models. There are some studies of DSMC techniques that describe the wetting effect by using an absorption probability (Frezzotti et al. 2014), but these models also require additional effort to find the right absorption probabilities for different gas–surface interactions. These empirical coefficients are hard to determine for complex systems.

Among many particle-based methods, MD offers molecular models that predict the diffusion coefficients most accurately. Besides, we already have developed a reliable water–silicon/silica interaction model for MD (Kim et al. 2014). Since the water–silicon/silica interaction model is an implicit model, the simulations can be performed much faster than by using other (explicit) MD models. Therefore, in this study we have chosen to use our implicit MD method to simulate the last stage of outgassing of water molecules from silicon/silica nanopores.

**Fig. 1:**
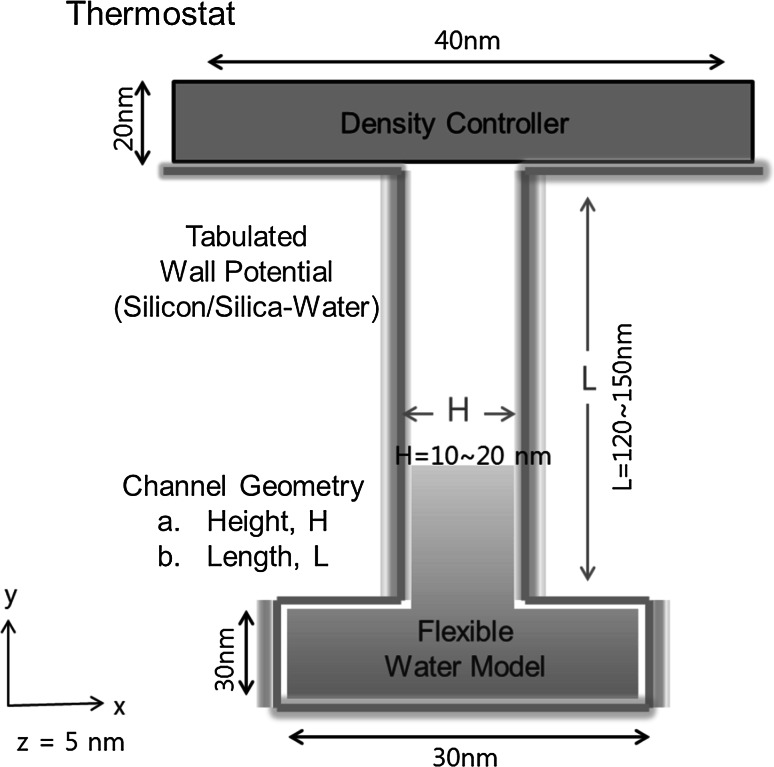
Overview of the nanopore and important factors for outgassing. On *top*, a density/pressure controller is used. The walls are modeled by wall potentials. The simulation system is 5 nm deep in *z*-direction. A periodic boundary condition is applied in $z$-direction

The molecular dynamics code PumMa (http://pumma.nl) has been used here. This code is validated and applied to nanoflow and heat transfer (Nedea et al. 2005; Akker et al. 2011, 2012; Kim et al. 2013). The nanopore for the outgassing simulation is modeled as a slit between two walls of parallel stacked silicon/silica plates extended from a cavity (Fig. 1). The simulation system is 5 nm deep in $z$-direction. Since a periodic boundary condition is applied in $z$-direction, it can be considered to have infinite depth in $z$-direction. The pore width $H$ is the distance between the silicon/silica walls of the nanopore. This pore width is chosen such that the potential energies of interaction exerted by the two opposing surfaces are not affected by each other. In our simulation, we use a minimum pore width of 10 nm.

To simulate the outgassing of water vapor from a silicon/silica nanopore, several molecular dynamics simulation techniques and models are used (Fig. 1): a pressure controller, a flexible three-point water model, and a water–silicon/silica tabulated wall potential.

**Fig. 2:**
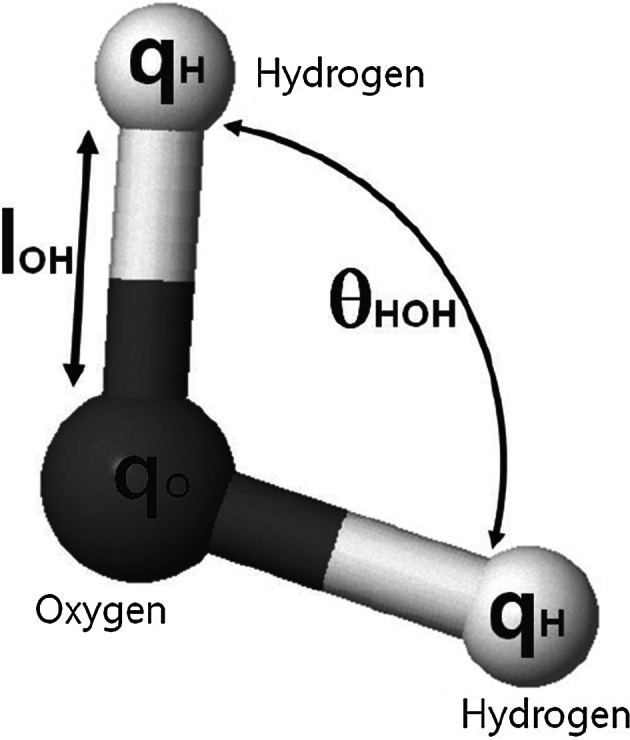
Flexible three-site water model

In this study, a modified flexible TIP3P water model is used (Fig. 2). The model is based on the model by Neria et al. (1996). It resembles that in all aspects, except for the molecular diameter of oxygen atom, $r_{0}^{{OO}}$. We use the same diameter that is used for the original TIP3P model ($r_{0}^{\mathrm{OO}}$ = 0.31506 nm) (Jorgensen et al. 1983). Parameters for the models are shown in Table 1.

**Table 1:** Nonbonded parameters, geometry, and electrostatic properties of the modified three-point water models

Parameter and unit		Original TIP3P (Jorgensen et al. 1983)	Mod. TIP3P (Neria et al. 1996)	Mod. TIP3P Kim
$r_{0}^{\mathrm{OO}}$ (nm)		0.31506	0.315365	0.31506
$\epsilon ^{OO}$ (kcal mol^−1^)		0.1521	0.1521	0.1521
$r_{0}^{\mathrm{HH}}$ (nm)		0	0.0449	0.0449
$\epsilon ^{\mathrm{HH}}$ (kcal mol^−1^)		0	0.046	0.046
$r_{0}^{\mathrm{OH}}$ (nm)		0	0.1993	0.1993
$\epsilon ^{OH}$ (kcal mol^−1^)		0	0.084	0.084
$q^{O}$ ($e$ units)		${-0.834}$	${-0.834}$	${-0.834}$
$q^{H}$ ($e$ units)		${-0.417}$	${-0.417}$	${-0.417}$
$l_{0}^{\mathrm{OH}}$ (nm)		0.09572	0.09572	0.09572
$\theta _{0}^{HOH}$(°)		104.52	104.52	104.52
$K_l$ (kcal mol^−1^ Å^−2^)		–	450	450
$K_{\theta }$ (kcal mol^−1^ rad^−2^)		–	55	55

Our results for the liquid density for modified TIP3P models are in good agreement with recent studies and have a slightly better radial distribution function than the model by Neria et al. (1996).

**Fig. 3:**
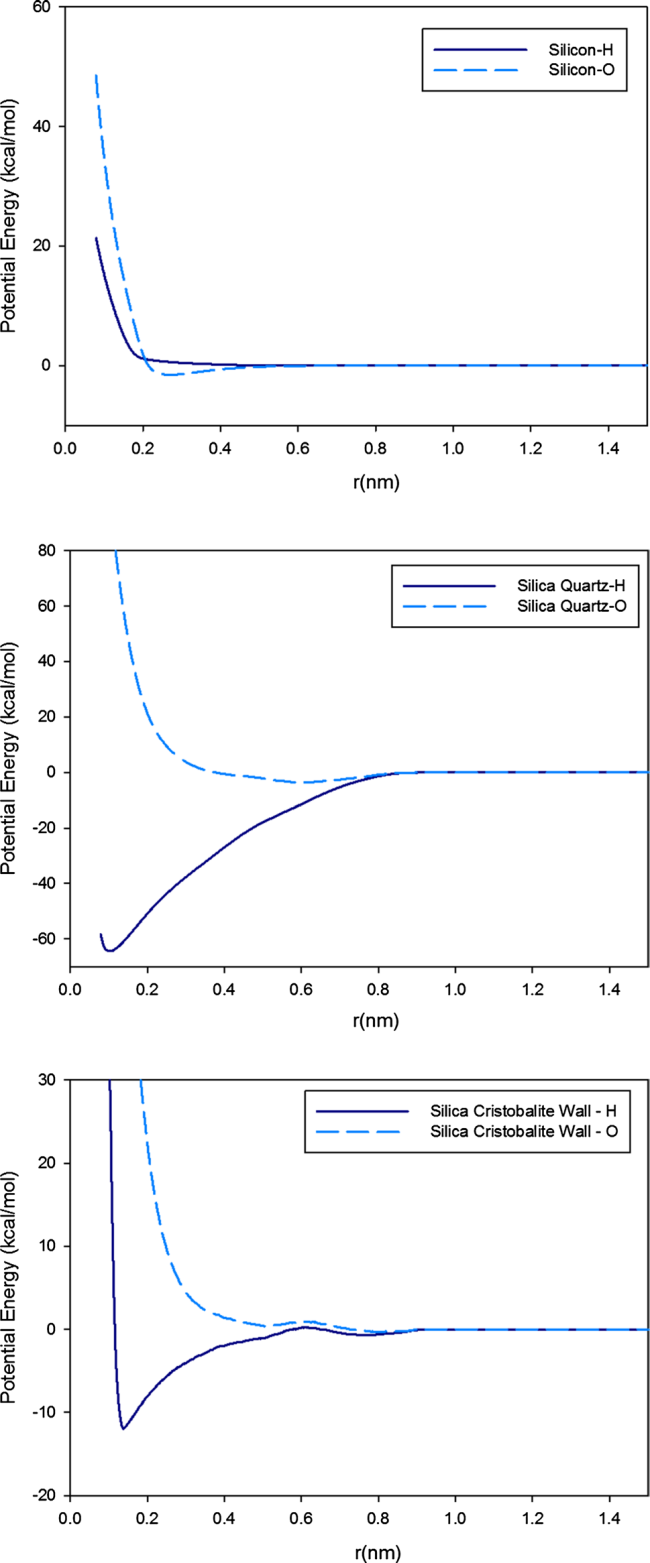
Tabulated wall potentials for silicon–water (*top*), $\alpha$ quartz–water (*middle*), and $\alpha$ cristobalite–water (*bottom*) (Kim et al. 2014). The lines correspond to the interaction between the wall and a single hydrogen or oxygen atom in the water molecule

The water–silicon/silica wall interactions are modeled by tabulated potentials as described in our recent paper (Kim et al. 2014). We improved the idea of the conventional wall potential so that it can describe the water–silicon and water–silica interactions. The new wall potential models a three-dimensional wall in the (1, 0, 0) plane. The wall potential is developed based on an EEM-based ReaxFF force field. Through several steps, a potential energy function for the wall–water interaction is tabulated (Kim et al. 2014). The resulting potential energy functions are shown in Fig. 3. The EEM method allows accurate electrostatic interaction of water and silica bulk since it accurately calculates the charge distribution of the system.

Since a smooth potential is employed to represent the silicon/silica planes, the wall is frictionless. This is not true for the silicon/silica atoms in a real adsorbent. In order to add friction-like behavior to the wall potential, the so-called diffuse boundary condition is employed. It is based on the diffuse boundary conditions used by Cracknell et al. (1995) and Travis and Gubbins (1999). However, it is modified here for the case of water molecules. Application of our molecular diffuse boundary condition proceeds as follows. After each molecular dynamics time step, we check to see whether the following two conditions are satisfied: (1) The center-of-mass momentum component of a given molecule in the perpendicular direction to the surface has reversed in sign compared to the previous time step. (2) The center of mass of that same molecule is within the repulsive region of the water–silicon/silica wall potential. When both conditions are satisfied, we reassign a new center-of-mass velocity component of that molecule. The molecule will keep its velocity component in the perpendicular direction to the surface in order to maintain the gas–wall interaction strength, while it gets a new velocity from the Maxwell–Boltzmann distribution at the appropriate temperature in the directions parallel to the surface to model a friction-like behavior.

Three different wall materials (silicon, silica-quartz, silica-cristobalite) are used as boundary of the nanopore to investigate the effect of materials on outgassing. Silica-cristobalite is used as a reference material for the simulations of other effects such as temperature, pressure, and geometric effects. Silica-cristobalite is chosen since it has an intermediate interaction strength among three materials; the contact angle simulation result for silicon is 129°, for silica-quartz it is 0°, and for silica-cristobalite it is 40° (Kim et al. 2014). Often the intermediate range of interaction strength is troublesome in the outgassing process (Jousten 1999).

The simulations are performed for water vapor at the temperature $T = 300$ K inside the cavity. The number of molecules for the bulk water vapor in equilibrium with the fluid is taken to be 1000 water molecules for all simulations. We have observed in our previous work (Kim et al. 2013) that continuum models break down near Kn =  0.4–0.5. Hence, we have calculated that about 1000 water molecules inside the cavity are needed for obtaining an initial Knudsen value of 0.4.

The simulations are carried out by varying the main factors of outgassing (Fig. 1): the outlet pressure, the temperature, the geometry of the nanopore, and the material of the nanopore. The Berendsen thermostat (Berendsen 1981) is used for controlling the system temperature in the whole domain. A thermostat is applied to the whole domain, since a temperature controlled oven is often used in real outgassing treatments.

The outlet pressure is controlled outside the nanopore (Fig. 1) by controlling the density of that outlet region. The density is controlled within the controller zone (outlet region) by deleting random molecules such that the correct number density is enforced. At time $t$, a controller computes the number of molecules that needs to be deleted. We track the remaining number of particles inside the system to compute the outgassing of water molecules from the silicon/silica nanopore.

To calculate a reliable outgassing result, we performed 6,000,000 time steps where one time step represents 0.0005 ps. The total simulation time is depending on the outgassing rate, since the computation time is directly related to the number of particles remaining inside the system. Therefore, most computational time is used in the early stage of the outgassing where a large number of particles are present in the pore. Simulations with fast outgassing rates take around 1 day on a single CPU core, while simulations with low outgassing rates take 2–3 days on a single CPU core.

## Results and discussion

### Effect of outlet pressure

In order to check the influence of the outlet pressure on outgassing, the outlet pressure was varied from 0 to 266 mbar. These outlet pressures cover perfect vacuum (0 mbar), medium vacuum (27, 80, 133 mbar), and low vacuum state (266 mbar). The chosen parameter values to analyze the influence of outlet pressure on outgassing are shown in Table 2.

**Table 2:** Parameter values for the study of the outlet pressure effect

Set 1: Varying outlet pressure	
Outlet pressure (mbar)	0, 27, 80, 133, 266
Temperature (K)	773 K
Channel width, *H* (nm)	10
Channel length, *L* (nm)	150
Surface material	Silica-cristobalite

**Fig. 4:**
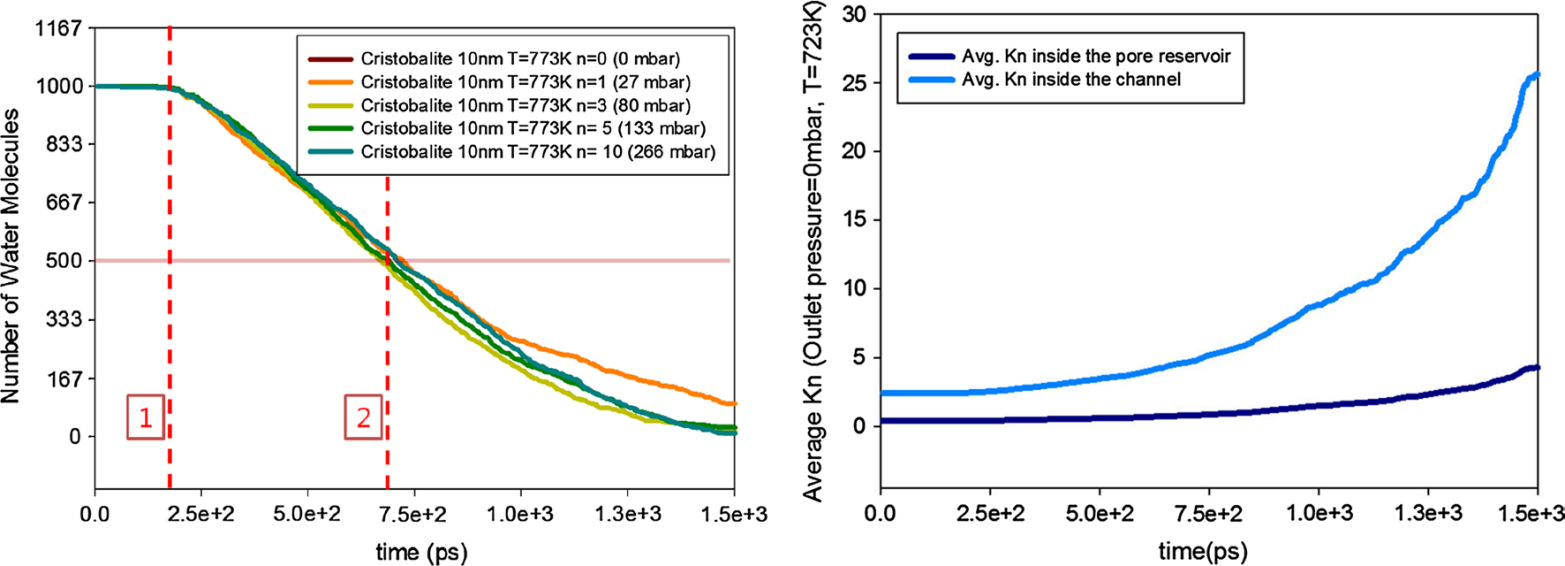
*Left* influence of outlet pressure on outgassing for a cristobalite nanopore with a channel width of 10 nm, a channel length of 150 nm, and outgassing temperature of 773 K. The first *dashed line* indicates the time for the water molecules to reach the outlet of the nanopore from their initial condition. The second *dashed line* indicates the half-life. *Right* change in average Knudsen number inside the cavity and the channel for an outlet pressure of 0 mbar

In real systems, where the pumping speed is constant, the pressure in the system will follow an exponential decay with time (Jousten 1999). Half-life is therefore used throughout the paper to represent the outgassing behavior.

In our study, half-life is defined as the time that is needed to reduce the amount of molecules to half of its initial value (Fig. 4, position 2). The half-life includes the time for the water molecules to reach the outlet of the nanopore from their initial positions (Fig. 4, position 1). For most of the outgassing studies, the time for the water molecules to reach the outlet of the nanopore is negligible compared to the experimental time. However, for our simulation scale, it is noticeable throughout the studies and also has an impact on the half-life: It changes by the outgassing factors. We do not divide the half-life into two different time sets as it is hard to distinguish the exact point where the outgassing phase changes. The half-lives of the simulations showed a standard error of 50 ps when 20 independent simulations are computed for $T=523$ K in a cristobalite nanopore with $H=10$ nm, $L=150$ nm.

The half-lives of water molecules for different pressures are shown in Table 3. These values are almost the same for all outlet pressures. Figure 4 (left) also shows that the decays in number of water molecules are nearly identical. This indicates that for $T = 773$ K, the outgassing rate is hardly influenced by the outlet pressure. The same trend is also observed for $T=523$ K. Figure 4 (right) shows the averaged Knudsen numbers for a outlet pressure of 0 mbar. It clearly shows that the gas flow starts in the transition regime and moves toward the free molecular regime. For the other outlet pressures, the graphs are similar.

**Table 3:** Half-lives of water molecules inside a silica-cristobalite nanopore for different outlet pressures

Outlet pressure	0 mbar	27 mbar	80 mbar	133 mbar	266 mbar
Half-life	0.71 ns	0.61 ns	0.67 ns	0.71 ns	0.79 ns

To explain this result, we have to look to the outgassing mechanism. The probabilities that the water molecules will interact with each other for all four pressures are too small to influence the outgassing process. The smallest mean free path for our simulations (at low vacuum state) is around 0.5 μm [based on an effective diameter of water vapor $=0.46$ nm (O’Hanlon 2003)]. This is more than 100 times bigger than the cutoff radius of our water–water interaction (2.5 nm) in our MD simulations. Hence, the outlet pressure will have a negligible influence on the outgassing for all simulation cases.

In conclusion, decreasing the outlet pressure by using a bigger pump size can be advantageous to enhance the outgassing, but not for the near vacuum state (perfect vacuum–low vacuum).

### Effect of temperature

Early workers baked systems to about 200 °C, which was effective in removing weakly bound surface water and hydrocarbon molecules (http://vacuumtunes.co.uk). The effectiveness of high baking temperature is demonstrated by Calder and Lewin (1967), who showed that outgassing could be reduced to about $10^{-16}$ mbar l/s cm$^2$ by baking for 11 days at 300 °C or only for 1 hour at 635 °C. In this section, the effect of temperature on outgassing is studied. Table 4 shows the parameter values for this study: *T* = 423, 523, 673, and 773 K. These test temperatures are chosen based on the studies by Ishikawa and Odaka (1990), Ishikawa et al. (1991) and Ishikawa and Yoshimura (1995)

**Table 4:** Parameter values for the study of the outlet temperature effect

Set 2: Varying temperature	
Outlet pressure (mbar)	0
Temperature (K)	423, 523, 673, 773
Channel width, *H* (nm)	10
Channel length, *L* (nm)	150
Surface material	Silica-cristobalite

**Fig. 5:**
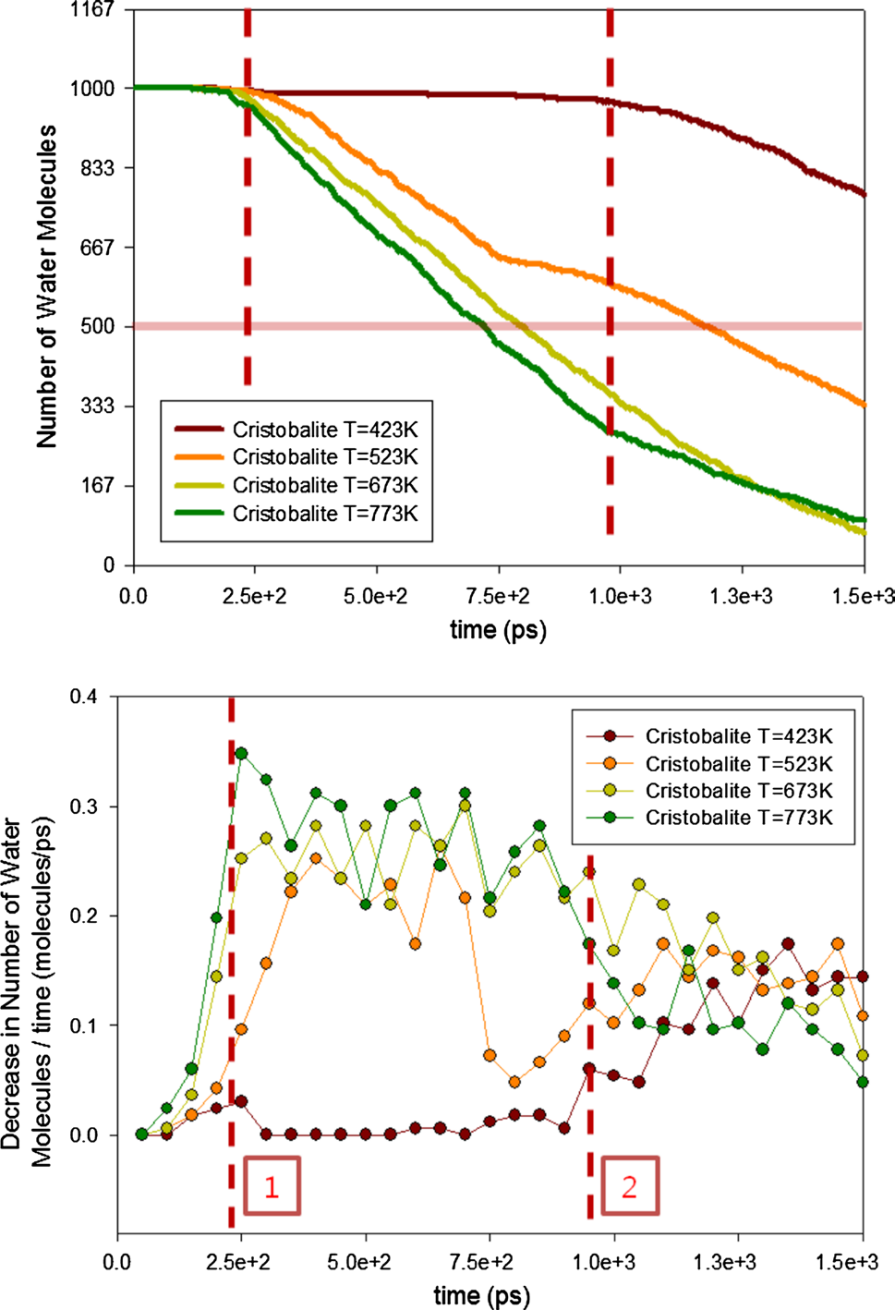
Influence of baking temperature on outgassing (*top*) and outgassing rate (*bottom*). Transparent horizontal *red line* shows the half-lives of the water molecules. *Dashed vertical lines* (*with numbers*) are explained in the text (color figure online)

The simulation results (Fig. 5 (top), and Table 5) clearly show the benefits of high baking temperatures and the diminishing amount of water molecules inside the nanopore with increasing baking time; the half-life of the water molecules is clearly shorter for higher temperatures.

**Table 5:** Half-lives of water molecules inside the nanopore for different temperatures

Temperature (K)	423	523	673	773
Half-life (ns)	2.05	1.18	0.81	0.72

Outgassing rate is shown in Fig. 5 (bottom) . It is defined as the decrease in the total number of molecules in the pore per unit of time. The simulation results show that temperature increases the outgassing rate the most when temperature changes from $T=423$ to $T=523$ K. The influence becomes minimal for higher temperatures. This is mainly because at the three higher temperatures ($T=523$, 673, and 773 K), water molecules need similar times to reach the outlet of the nanopore (Fig. 5, position 1), while at $T=423$ K, it requires much longer time (Fig. 5, position 2).

According to Hässig et al. (2011), the outgassing process can be explained with three different mechanisms: desorption, diffusion, and decomposition.

By theory, desorption outnumbers the effect of diffusion greatly in the initial phase of outgassing. The rate of desorption will depend on the binding energies of the gas–wall interaction, the surface temperature, and the surface coverage (Jousten 1999). If all absorbed molecules have been desorbed from the surface, a different mechanism of outgassing comes into action (Jousten 1999). Water molecules dissolved in the bulk should diffuse to the region where the concentration is lower. The outlet of the nanopore is such a region for our simulations. Eventually, it will lead to outgassing.

In our simulations, desorption and diffusion both take place at the same time. A water molecule which is adsorbed on the surface will desorb from the surface, and will diffuse toward the outlet of the nanopore. But it will eventually re-adsorb again while it is flowing out.

When the outgassing process is diffusion limited, it can be described by Fick’s diffusion equation (Fick 1855):

1 $${\hbox {Outgassing rate}} = Q A,$$

2 $$\begin{aligned} Q= & {} -D \frac{\hbox {d}c}{\hbox {d}x}, \end{aligned}$$

where $Q$ is the diffusion flux, $A$ is the area which the particles diffuse through, $c$ is the concentration, and $D$ is the diffusion coefficient, which depends on temperature. However, it is not easy to find a correct diffusion coefficient, especially, since it is also influenced by its surrounding (e.g., Knudsen diffusion is dependent on surface material). Therefore, we decided to compare our MD results with the outgassing rate computed by several diffusion models from the literature.

Firstly, the diffusion coefficient was calculated based on the equation by Chapman and Cowling (1970):

3 $$\begin{aligned} D_C= \frac{3}{8 n d^2} \left( \frac{k T}{\pi m}\right) ^{1/2} \end{aligned}$$

where $n$ is the number density, $d$ is diameter of the molecule, and $m$ is its mass. The molecular diameter of a water molecule is $d=0.29$ nm and its mass $m = 18$ g/mol, resulting in a diffusion coefficient $D_C = 13.4 \cdot 10^{-6}$ m^2^/s.

Secondly, the diffusion coefficient was calculated based on the equation by Atkins (1994):

4 $$\begin{aligned} D_A = \frac{1}{3} \lambda \left( \frac{8 k T}{\pi m}\right) ^{1/2} \end{aligned}$$

where $\lambda$ is the mean free path. If we use for the Knudsen diffusion a simple model that excludes the effect of the surface material, the mean free path length $\lambda$ can be replaced by the channel width $H=10$ nm, resulting in an effective diffusion coefficient $D_{A}= 5.23 \cdot$ 10^−6^ m^2^/s.

Thirdly, the diffusion coefficient of water vapor can also be calculated based on the empirical relation derived by Bakhtar and Zidi (1985):

5 $$\begin{aligned} \frac{D_B}{D_D} = \frac{\sqrt{(T/T_{D})}}{(p/p_D)^{n_B}} \end{aligned}$$

where $D_D = 0.104 \cdot 10^{-6}$ m^2^/s at $p_D = 1$ bar, $T_D=373.15$ K, and $n_B=0.868873$. With an averaged pressure $p=6.5$ bar (the pressure inside the cavity is estimated to be $p=13$ bar, based on ideal gas law and $p \approx 0$ bar at the outlet), and $T=523K$, the diffusion coefficient is $D_B = 2.42 \cdot$ 10^−6^ m^2^/s

**Fig. 6:**
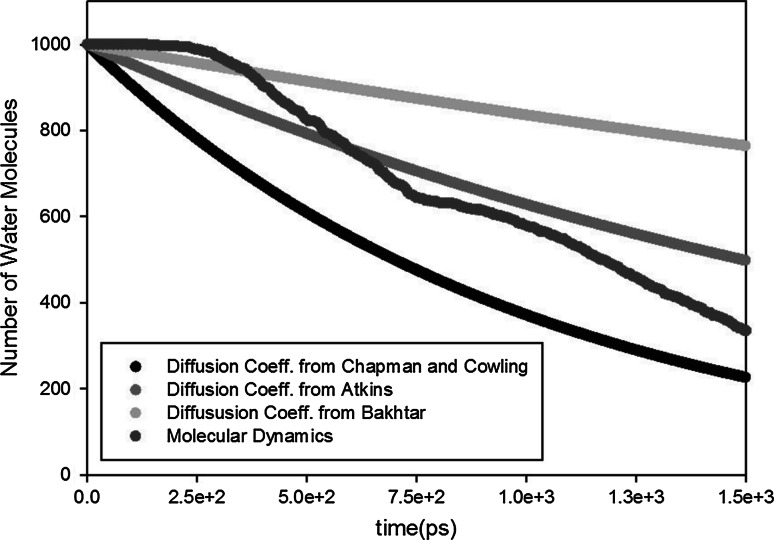
Comparison between our molecular dynamics simulation of a pore made of cristobalite ($H=10$ nm and $L=150$ nm) and diffusion-based continuum models at $T=523$ K

Figure 6 shows the decay in the number particles according to these three diffusion models. Due to the large variation in diffusion coefficients, different slopes can be observed. The MD results for cristobalite are in between these lines. But its shape a slightly different: There is no pure exponential decay. For example, a kink in the MD graph can be observed at $t \approx 750$ ps. This indicates that in the beginning of the outgassing process, some part of the water molecules that are initially in the middle of the pore will be solely diffuse to the outlet without interacting with the channel walls. At a certain moment, more water molecules will interact with the walls before reaching the outlet and the adsorption/desorption process will slow down the outgassing process. This effect will be smaller for high temperatures because then the adsorption/desorption process is faster (Fig. 5).

**Fig. 7:**
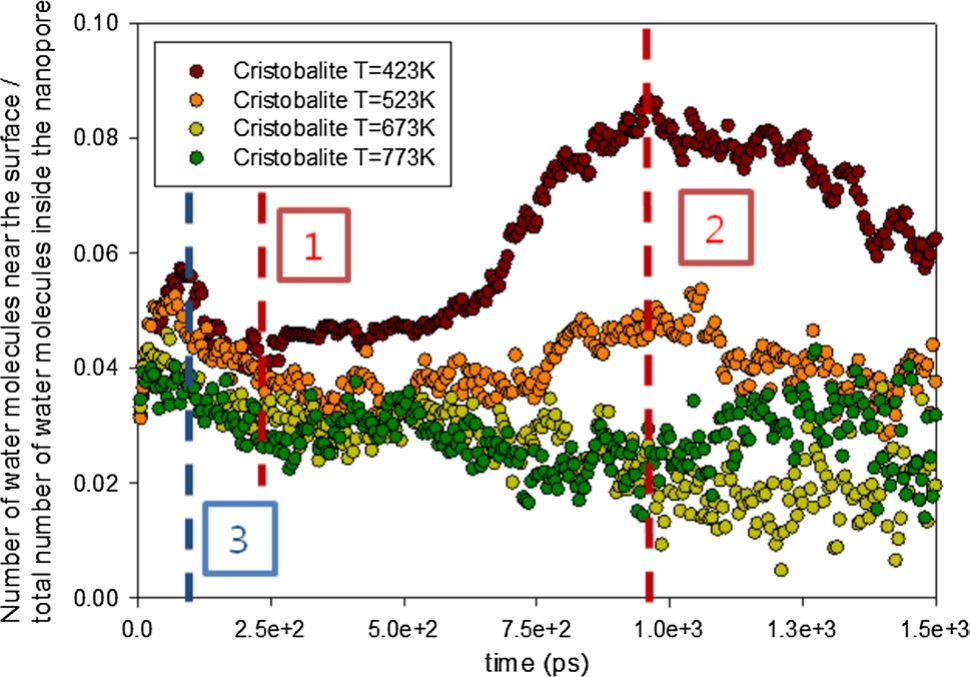
Distribution of the water molecules in the nanopore. Positions *1* and *2* are also shown in Fig. 5. The *dashed vertical lines* (*with numbers*) are explained in the text

In order to study the absorption/desorption effect at different temperatures, we have calculated the ratio of the number of water molecules that are near the wall surface and the total number of water molecules in the nanopore (Fig. 7). A molecule which has a distance less than $2.5$ nm from the wall surface is counted as a water molecule near the surface: The rest is considered to be in the bulk. A distance of $2.5$ nm is chosen since it is the cutoff radius for the tabulated wall potentials; there will be no direct interaction anymore when the distance between the molecule and the wall is larger than this cutoff distance.

Based on the density ratio, the desorption rate is considerably smaller for $T=423$ K than for other temperatures inside the silica-cristobalite nanopore. It is shown that nearly two times more water molecules are near the surface for $T=423$ K than for higher temperatures.

In Fig. 7, there are two positions where the density of the molecules near the surface increases. The first increase (from $0$ ps to position 3) can be due to a change in position of the water molecule due to the system equilibration. In the initial state, water molecules have been randomly placed inside the cavity of the nanopore. However, when the simulation is started, water molecules are attracted by the surface due to the interaction with the wall potential. The second increase (from $7500$ ps to position 2) only happens at $T=423$ K. This increase can be explained by the removal of bulk particles. If the desorption rate is limiting the outgassing of water molecules, the bulk particles will be flowing out easier than the particle absorbed on the surface where friction exists. Hence, it will be the bulk particle to be the first to reach the outlet of the nanopore. Therefore, the ratio increases rapidly. However, this increase does not appear for higher temperatures (position 1). It indicates that for high temperatures, desorption rates are high enough such that even water molecules near the surface are hardly influenced by the friction of the wall surface.

After position 1 (for $T=523$, 673, and 773 K) and position 2 (for $T=423$ K), the number of molecules inside the cristobalite nanopore decreases and the pressure of gas inside the nanopore decreases at constant temperature. According to the adsorption isotherm theory (Langmuir 1916; Brunauer et al. 1938) which explains the adsorption of gas molecules on a solid surface, the quantity of gas adsorbed on a given surface decreases when the pressure decreases. We observe that the ratio decreases smoothly with time, since the pressure (related to the total number of water molecules) inside the nanopore is decreasing with time as well.

### Effect of geometry

The geometry effects on outgassing are indicated by several researchers. Ishikawa and Odaka (1990), Ishikawa et al. (1991), Ishikawa and Yoshimura (1995) indicated that surface treatments and raw material quality (like surface roughness) can improve the outgassing rate substantially. It was also found by Tuller et al. (1999) that water sometimes get stuck during the outgassing at some specific locations, such as a corner.

The geometry of the nanopore is studied in order to determine how the crack size on the surface or the geometry of the MEMS/NEMS device can influence the outgassing. The geometry of the nanopore is described by two variables which are shown in Fig. 1: channel width $H$ and channel length $L$.

#### Channel width

Two channel widths ($H = 10$ and $20$ nm) are tested at four different temperatures as described in Table 6.

**Table 6:** Parameter values for the study of the geometry effect (channel width)

Set 3a: Varying channel width	
Outlet pressure (mbar)	0
Temperature (K)	423, 523, 673, 773
Channel width, *H* (nm)	10, 20
Channel length, *L* (nm)	150
Surface material	Silica-cristobalite

**Fig. 8:**
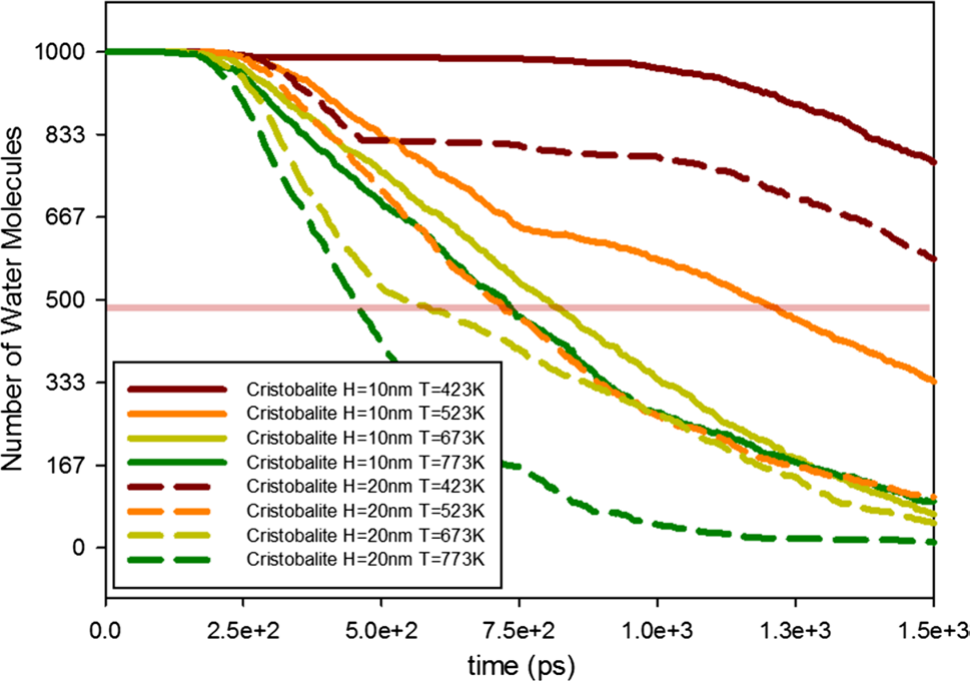
Influence of channel width on outgassing. Transparent horizontal *red line* shows the half-lives of the water molecules (color figure online)

As shown in Fig. 8, the channel width clearly has an influence on the outgassing of water molecules; the larger the channel width is, the faster the outgassing is. By increasing the channel width from $H = 10$ to $H = 20$ nm, half-life of the water molecules is decreased by a factor of around 0.63 for temperatures above 523 K (Table 7). For 423 K, the factor even increases to 0.8.

**Table 7:** Half-lives of water molecules inside the nanopore for different channel widths

	Temperature (K)	423	523	673	773
$H=10$ nm	Half-life	2.05 ns	1.18 ns	0.81 ns	0.72 ns
$H=20$ nm	Half-life	1.63 ns	0.71 ns	0.54 ns	0.46 ns
Factor	Half-life (20 nm)/half-life (10 nm)	0.80	0.60	0.67	0.65

From these results, we can conclude that the effect of larger width size is more dominant for higher temperatures. A similar effect occurs for lower temperatures in the beginning of the outgassing, but eventually the outgassing rate becomes similar to the one for smaller channel widths. Increasing the channel area will increase the outgassing by diffusion. When the outgassing process is diffusion limited, it can be described by Fick’s diffusion equation (Fick 1855). Equation (1) shows that outgassing is directly proportional to the cross-sectional area of the channel. Therefore, the effect of larger width size will be more influential to the outgassing by diffusion than outgassing by desorption. In addition, the effect will be more dominant for higher temperatures as mentioned in Sect. 3.2.

#### Channel length

**Fig. 9:**
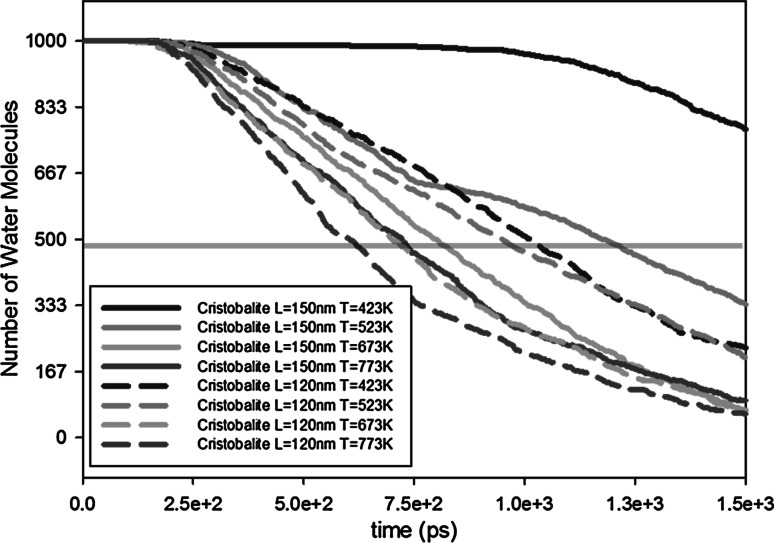
Influence of channel length on outgassing. Transparent horizontal *red line* shows the half-lives of the water molecules (color figure online)

Two channel lengths ($L = 120$ and 150 nm) are simulated at four different temperatures as described in Table 8. As shown in Fig. 9, the channel length also has an influence on the outgassing of water molecules; the shorter the channel length is, the faster the outgassing occurs. By decreasing the channel length, the half-life of water molecules has decreased by a factor of around 0.84 for temperatures above 523 K (Table 9). For 423 K, the factor is smaller and is 0.5. From these results, we can conclude that the effect of shorter channel length is more dominant for lower temperatures. This result is in line with the earlier observation that desorption has a larger influence for lower temperature. By reducing the channel length, we reduce the surface area which reduces the chance of absorption. Eventually, this will reduce the effect of desorption on the outgassing.

**Table 8:** Parameter values for the study of the geometry effect (channel length)

Set 3b: varying channel length	
Outlet pressure (mbar)	0
Temperature (K)	423, 523, 673, 773
Channel width, *H* (nm)	10
Channel length, *L* (nm)	150, 100
Surface material	Silica-cristobalite

Furthermore, if the diffusion process would be dominant, the ratio between the half-life of the 120 nm and the 150 nm channels should have been constant and equal to 0.64 since the diffusion time goes quadratically with the diffusion length. This is not observed in Table 9.

**Table 9:** Half-lives of water molecules inside the nanopore for different lengths

	Temperature (K)	423	523	673	773
*L* = 120 nm	Half-life	1.02 ns	0.96 ns	0.71 ns	0.61 ns
*L* = 150 nm	Half-life	2.05 ns	1.18 ns	0.81 ns	0.72 ns
Factor	Half-life (120 nm)/half-life (150 nm)	0.50	0.81	0.87	0.84

For a cristobalite nanopore, we can conclude that the channel length is more influential for the outgassing at lower temperature (where desorption is important), while the channel width is more influential for the outgassing at higher temperatures (where desorption is less important).

### Effect of materials

All solid surfaces have attraction forces normal to the surface. So molecules landing on the surface can be adsorbed by the wall material. The absorbed molecules can act as the main source of contaminant gas in vacuum systems. Adsorption takes place by physical adsorption or chemical adsorption (De Segovia 1999). In physical adsorption, gas molecules are attracted weakly by van der Waals forces with binding energies of less than 10 kcal/mol, while in chemisorption, actual chemical bonding occurs between the gas molecules and the molecules and atoms on the surface of the vacuum material. Chemisorption has a binding energy more than 20  kcal/mol.

**Table 10:** Parameter values for the study of the material effect

Set 4: various materials	
Outlet pressure (mbar)	0
Temperature (K)	423, 523, 673, 773
Channel width, $H$ (nm)	10
Channel length, $L$ (nm)	150
Surface material	Silica-cristobalite, silica-quartz, silicon

Three different surface materials (silica-cristobalite, silica-quartz, silicon) are simulated for four different temperatures as described in Table 10. Binding energy for the water–silicon, water–quartz, and water–cristobalite tabulated wall potential is 1  kcal/mol, 60  kcal/mol, and 10 kcal/mol (Kim et al. 2014). This cannot be directly compared to the binding energies of physical adsorption ($\sim$10 kcal/mol) and chemical adsorption ($\sim$20 kcal/mol) values since they are the sum of multiple interactions between a gas molecule and multiple wall atoms, but they still give a clear indication on the type of water–wall interactions.

Since we did not take reactions into account for the tabulated wall potentials, all binding energies are not due to chemical adsorption. The strong binding energy might be due to the strong hydrogen bonding. Normal hydrogen bonds are regarded as those with strengths of about 3–5 kcal/mol and are generally less than 12 kcal/mol. However, some strong hydrogen bonds may have energies in excess of 12 kcal/mol (Chen et al. 1998).

**Fig. 10:**
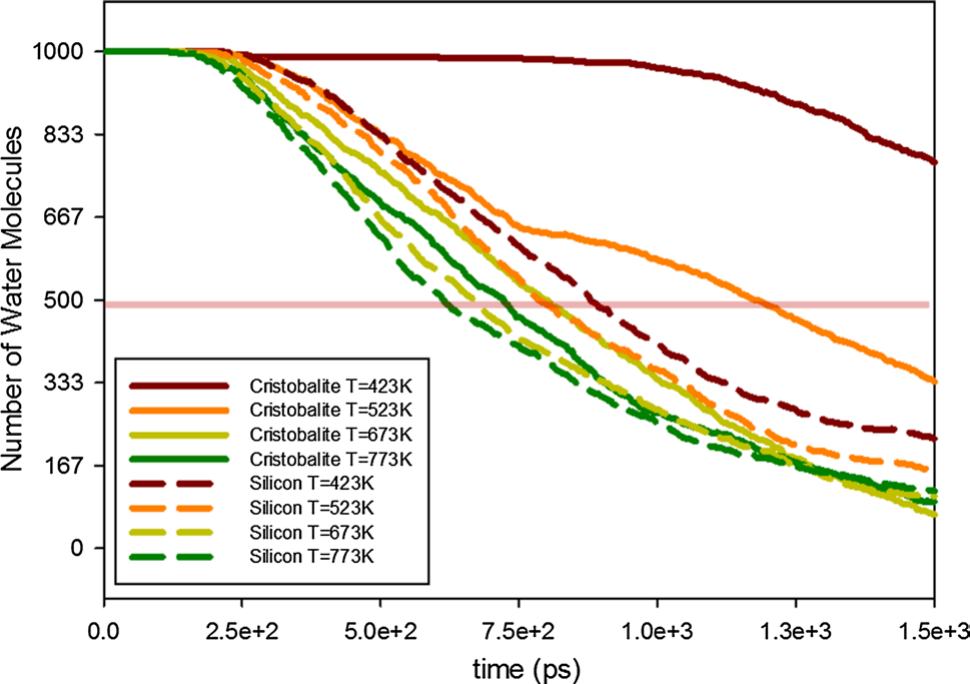
Influence of nanopore material on outgassing. The transparent horizontal *red line* shows the half-lives of the water molecules (color figure online)

Figure 10 shows the outgassing of water molecules in different nanopores (silicon and silica-cristobalite), while Table 11 shows the half-life of the water molecules inside the nanopore for different materials. The results for silica-quartz are not shown in Fig. 10 and Table 11 because it did not show any outgassing within the simulation time. Table 11 also shows the effect of different materials as the temperature increases: Half-life of the water molecules is increasing by a factor (silicon/cristobalite) of around 0.43 for temperature $T = 423$ K to a factor of 0.86 for $T = 773$ K

**Table 11:** Half-lives of the water molecules inside the nanopore for different materials

	Temperature (K)	423	523	673	773
Cristobalite	Half-life	2.05 ns	1.18 ns	0.81 ns	0.72 ns
Silicon	Half-life	0.89 ns	0.79 ns	0.67 ns	0.62 ns
Factor	Half-life ($silicon$)/Half-life ($cristo.$)	0.43	0.67	0.82	0.86

Two main interpretations can be made on the basis of these results. For low binding energy, the desorption rates are so high that the corresponding molecules quickly desorb from the wall surface and cause no further problems in outgassing. For very high values of binding energy, the desorption rates are too small to see any outgassing. Silica-quartz is an example of a case of very high binding energy and it is observed that the outgassing is hardly influenced by the temperature. Once water molecules are adsorbed on the surface, they cannot overcome the binding energy of the silica-quartz surface and stay there; a thin water layer is formed on the surface. Hence, no outgassing happened within the simulation time.

An additional simulation has been made with $T = 1500$ K, but even for this high temperature, the simulation of silica-quartz shows that the temperature has no influence on the outgassing rate. However, in industry, the baking temperature often used for silica material is around $1000$ °C Akermark et al. (1996). This difference between experiment and simulation might be coming from neglecting the change in crystal structures at high temperatures. Silica materials are known to change its structure from silica-quartz to silica-cristobalite when the temperature increases. According to the study of Heaney (1994) and Heaney et al. (1994), silica-quartz transforms to silica-cristobalite at a temperature of 1050 °C. As observed, silica-cristobalite allows water molecules to outgas in a reasonable time. The temperature of T = 1000 °C, which is used in industry, might be the temperature needed for transformation of the surface rather than the temperature needed to overcome the binding energy.

## Conclusion

In this paper, the main factors affecting outgassing of water molecules are studied by varying the system temperature, the outlet pressure, the geometry of the nanopore, and the materials of the nanopore. A negligible influence of outlet pressure on the outgassing rate is found (for pressure range up to low vacuum), while the temperature plays an important role in the outgassing process. By increasing the system temperature from 423 to 773 K, the half-life of the water vapor decreased with a factor of about 0.63 for the silicon channel and a factor of 0.35 for the silica-cristobalite. Two different outgassing mechanisms (desorption and diffusion) are discussed together with the effect of temperature on outgassing rate. The geometry of the channel also has an influence on outgassing rate, but not as much as the surface material: An increase in channel height from 10 to 20 nm results in a decrease in half-life of water vapor with a factor of about 0.8 for 423 K and 0.6 for 773 K. For the silica-quartz nanopore, formation of a thin water film on the surface is observed due to its very high binding energy, and hardly any outgassing of water vapor is found.
